# Lower myocardial perfusion in borderzones in the area at risk than out of the area at risk in acute myocardial infarction

**DOI:** 10.1186/1532-429X-17-S1-P128

**Published:** 2015-02-03

**Authors:** Kaatje Goetschalckx, Frank E Rademakers, Jan Bogaert, Attila Toth, Béla Merkely, Stefan Janssens, Piet Claus

**Affiliations:** Cardiovascular Diseases, University Hospital Leuven, Leuven, Belgium; Medical Imaging Research Center, University Hospitals Leuven, Leuven, Belgium; Department of Cardiovascular Sciences, KU Leuven, Leuven, Belgium; Department of Radiology, University Hospitals Leuven, Leuven, Belgium; Heart and Vascular Center, Semmelweis University, Budapest, Hungary

## Background

Myocardial infarction (MI) leads to heart failure in a substantial number of patients. Investigation of perfusion in the infarct-adjacent segments can offer better insights into left ventricular remodeling. It is the aim of this study to compare myocardial blood flow (MBF) with cardiac magnetic resonance (CMR) in the infarct borderzones (BZ) and the remote myocardium (RM) in a large patient population in the acute phase of MI.

## Methods

In this substudy of the NOMI-trial (ClinicalTrials.gov identifier: NCT01398384) using the placebo arm, 68 patients underwent CMR with restperfusion within 48 to 72 hours of acutely revascularized ST-elevation MI in a 1.5 tesla MR scanner (Achieva, Philips Medical Systems, The Netherlands). TIMI 2-3 flow was achieved in all patients after PCI. MBF was quantified using Fermi deconvolution with a dual bolus analysis technique (equal volumes of 0.0027 mmol/kg followed by 0.05 mmol/kg after a 20-s pause of contrast agent (Dotarem, Gd-DOTA, Guerbet, France)) in basal and midventricular short axis perfusion slices. A segmental model was applied on corresponding images of perfusion, late gadolinium enhancement (LGE) and T2-weighted images (T2W). The MBF of the infarct zone (IZ) was calculated as the mean perfusion of segments with LGE. The adjacent 30 degree segments were defined as BZ, and were also classified in BZ-inAAR and BZ-outAAR, depending on their location in- or outside the area at risk, respectively, as determined by T2W imaging. The MBF of the RM was calculated as the mean perfusion of the remaining segments. Apical slices seldomly included more than one myocardial zone and were excluded from analysis.

## Results

One hundred thirty six slices were analyzed. Restperfusion was not significantly different in BZ and RM (resp. 0.50 ± 0.22 and 0.51 ± 0.23 ml/g/min, p = 0.664). BZ-inAAR restperfusion however, was significantly lower than BZ-outAAR and RM perfusion (resp. 0.45 ±.19, 0.54 ±.24 and 0.51 ±0.23 ml/g/min, p < 0.05) in the acute phase of MI. (cf. Table)

**Table 1:** Table 1

MBF(ml/g/min)	IZ	BZ-inAAR	BZ-outAAR	RM
n =	114	95	116	130
rest perfusion	**0,37 Â± 0,19**	0,45 Â± 0,19*	0,54 + 0,24*	0,51 Â± 0,23 ns*

* p < 0.05

## Conclusions

Myocardial restperfusion in infarct-adjacent segments is lower in- than outside the area at risk in acute MI. This regional myocardial perfusion inhomogeneity may impact subsequent LV remodeling, thereby offering new opportunities for targeted interventions.

## Funding

No disclosures.

**Figure 1:**
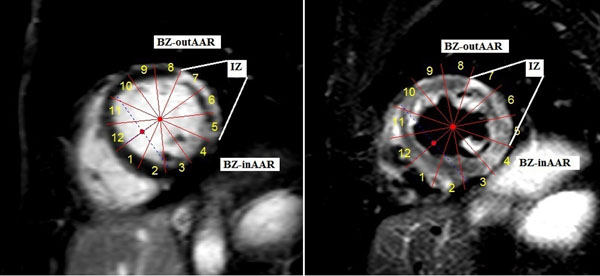
The left figure shows the late gadolinium enhancement midventricular short axis slice of an acute phase MI, with segments 5-7 defined as IZ (including microvascular obstruction), segments 4 and 8 as BZ and the remaining segments as RM. The right figure shows the corresponding T2-weighted image, with segment 4 defined as BZ-inAAR, and segment 8 as BZ-outAAR.

